# Prosthodontic Management of Combination Syndrome Using Digital Scanning and Direct Metal Laser Sintering Crowns: A Novel Approach

**DOI:** 10.7759/cureus.80124

**Published:** 2025-03-06

**Authors:** Shruti Jumde, Tushar Tanwani, Gaurav Tripathi, Keerthi Rohini, Anindita Chakraborty, Shubham Tale

**Affiliations:** 1 Department of Prosthodontics, Crown, and Bridge, New Horizon Dental College and Research Institute, Bilaspur, IND; 2 Department of Periodontology and Implantology, Government Dental College and Hospital, Mumbai, IND

**Keywords:** combination syndrome, complete denture, edentulism, mandibular distal extension prosthesis, removable partial denture

## Abstract

Combination syndrome presents unique challenges in prosthetic rehabilitation due to severe maxillary ridge resorption, supra-eruption of mandibular anterior teeth, and occlusal imbalances. This case report describes the management of a 76-year-old male patient with an edentulous maxilla and partially edentulous mandible, rehabilitated using a conventional maxillary complete denture and a mandibular removable partial denture since the patient declined implant-based treatment due to financial and surgical constraints. A mucostatic impression technique was used to avoid displacing hyperplastic tissues, ensuring optimal denture adaptation. To enhance durability and occlusal stability, direct metal laser sintering (DMLS) crowns were incorporated into the denture design to help minimize attrition and maintain occlusal harmony. The prosthesis was modified to evenly distribute the occlusal forces to prevent further ridge resorption and improve functional efficiency. The patient reported significant improvement in mastication, speech, and aesthetics, with no complications observed at the 12-month follow-up. The present case report highlights the importance of biomechanical considerations in managing combination syndrome. It also demonstrates that a well-planned, non-implant-based prosthetic approach can offer a stable, functional, and cost-effective solution for patients with financial or surgical limitations.

## Introduction

Combination syndrome is a peculiar dental condition wherein the maxilla is completely edentulous, and the mandible is partially edentulous with only anterior teeth present [1]. The condition was first reported by Ellsworth Kelly in 1972, and presently, it occurs in 24% of denture patients [2,3]. The discrepancy between the dentition of both arches precipitates a cascade of osseous and mucosal transformations in the oral cavity, such as an anterior maxillary ridge, hypertrophy of the maxillary tuberosities, papillary hyperplasia of palatal tissues, extrusion of mandibular anterior teeth, and resorption beneath mandibular partial denture bases [4]. These alterations compromise functioning and esthetics, significantly affecting the oral health-related quality of life of the individual.

One of the critical factors compromised in combination syndrome is the maintenance of the vertical dimension of occlusion (VDO). The loss of posterior occlusion results in an increased occlusal load on the anterior teeth, leading to their supra-eruption and further disruption of the occlusal plane. Additionally, excessive forces on the edentulous maxilla accelerate bone resorption, further exacerbating the loss of vertical dimension [4]. This reduction in VDO leads to functional impairments such as altered phonetics, masticatory inefficiency, and neuromuscular imbalance, significantly affecting the patient's overall prosthetic prognosis. Therefore, a fundamental goal in the management of combination syndrome is to restore and maintain an appropriate vertical dimension to improve function and preserve the remaining structures.

Dental rehabilitation in such patients is extremely challenging as it is complicated by severe tissue alterations in the oral cavity. A comprehensive understanding of the etiology of bone loss in combination syndrome is crucial for effective treatment planning. The primary reason for bone resorption in such cases is the uneven force distribution due to the presence of only anterior mandibular teeth, which results in excessive compressive and tensile stresses on the maxillary anterior ridge. These forces accelerate bone loss, leading to progressive ridge atrophy despite denture rehabilitation [5]. Additionally, factors such as occlusal overload, parafunctional habits, poor prosthetic design, and systemic conditions like osteoporosis or diabetes can further aggravate bone resorption in these patients.

It has been suggested that compressive forces are well tolerated by edentulous ridges while shearing forces are detrimental to long-term prosthodontic rehabilitation [6]. Prostheses with improper designs exert excessive forces that lead to residual ridge resorption. A well-designed prosthesis will exert intermittent forces of moderate intensity that can stimulate and preserve the bony ridge. A fundamental principle in prosthodontics is, therefore, to fabricate denture bases with broad coverage of denture bases to distribute forces over a larger area that reduces pressure per unit area [7].

In some cases, bone loss occurs even though the prosthesis is mechanistically well-designed. The influence of local and systemic factors has to be taken into account in such cases. Particularly, in the case of combination syndrome, the maxillary residual ridge destruction induced by the retained mandibular anterior teeth remains prevalent even after rehabilitation by a denture [4]. Advancements in digital dentistry provide improved solutions for such cases, offering enhanced precision in prosthesis fabrication and better occlusal adjustments through digital scanning and computer-aided design (CAD) techniques. One such innovation is direct metal laser sintering (DMLS) crowns, which provide superior strength, marginal adaptation, and long-term durability compared to conventional cast restorations, making them a viable option for restoring occlusal stability in combination syndrome cases.

Management of such challenges requires a comprehensive understanding of the biomechanical interactions between prosthetic devices and the oral environment. The present case report demonstrates a novel approach to address issues with the rehabilitation of a patient with combination syndrome while improving functional and aesthetic outcomes. This approach involves customization of the posterior occlusion in such a way that it allows a better distribution of occlusal forces, improving the stability of the denture, and preventing further bone loss or damage to the remaining natural teeth.

## Case presentation

A 76-year-old male patient reported to the institutional department of prosthodontics of the Pandit Deendayal Upadhyay Memorial Health Science and Ayush University of Chhattisgarh in Uparwara, India, in January 2024. The patient complained of a loose maxillary complete denture. He also complained of difficulty in chewing and speaking clearly for a year. No relevant history of medical conditions or known drug allergies was elicited.

**Figure 1:**
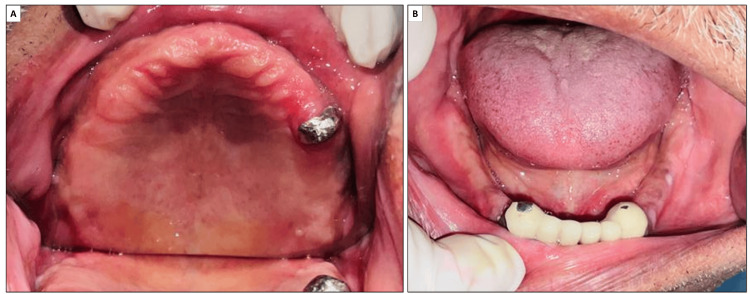
Intraoral examination revealed a completely edentulous maxilla (A) and a partially edentulous mandible (B).

On clinical examination, a completely edentulous maxillary arch and a partially edentulous mandibular arch with only mandibular anterior teeth were observed (Figure 1). A substantial amount of ridge resorption was observed in the maxillary anterior region. The mandibular teeth appeared to be supra-erupted. There was also an overgrowth of maxillary tuberosities observed bilaterally. Given the clinical features, the patient was diagnosed with combination syndrome.

The case could be managed by two treatment strategies. The first involved an implant-supported prosthesis, wherein a suitable number of implants would be placed across the maxillary arch, followed by a denture coverage. The second comprised a conventional complete denture, which was, however, challenging because of the prominent ridge resorption and focal hyperplastic tissues. The patient was reluctant to undergo multiple surgical procedures required for implant placement and was also unable to financially afford the former treatment option. Thus, he opted for a conventional complete denture in the maxillary arch and a removable partial denture (RPD) in the mandibular arch. The complete treatment plan was thoroughly explained to the patient, along with the challenges, limitations, post-treatment expectations, and possible complications. Informed consent was then obtained from the patient.

A preliminary impression of the maxillary arch was made using an impression compound (Rolex, Ashoo Sons, New Delhi, India), while that of the mandibular arch was made using irreversible hydrocolloid material (Zhermack, Badia Polesine, Italy). Both impressions were poured with type II dental stone (Kalabhai Kaldent, Mumbai, India) to create diagnostic casts for further steps of denture fabrication.

**Figure 2:**
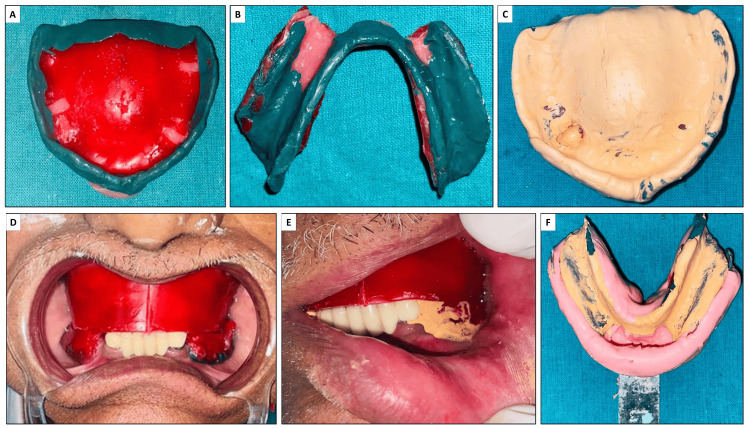
(A) and (B) Border molding of maxillary and mandibular base plates; (C) Final maxillary impression; (D)–(F) Final mandibular impression using McLean’s impression technique.

A custom tray was fabricated with a wax relief placed over the anterior hypermobile tissue to ensure proper adaptation and minimize tissue distortion. Border molding was performed using a green modeling compound (Dental Products of India, New Delhi, India) to capture the functional extent of the tissues accurately. The final impression for the maxillary arch was made using zinc oxide eugenol impression material (Dental Products of India). For the mandibular arch, McLean’s impression technique was utilized (Figure 2), and the impression was subsequently poured using type III dental stone (Kalabhai Kaldent) to create a precise cast.

**Figure 3:**
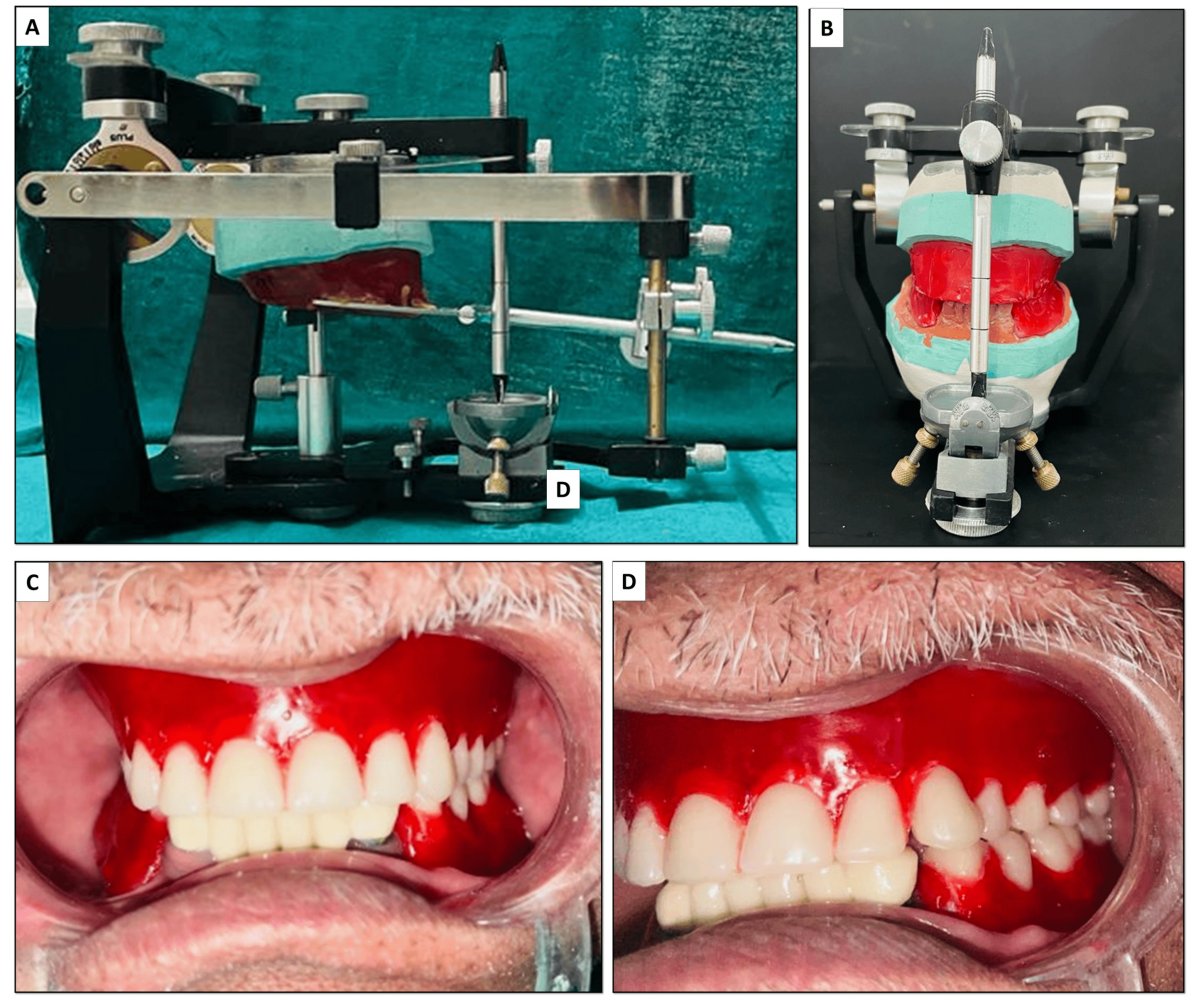
(A) and (B) Facebow transfer and mounting on a semi-adjustable articulator; (C) and (D) Denture try-in after tooth arrangement

**Figure 4:**
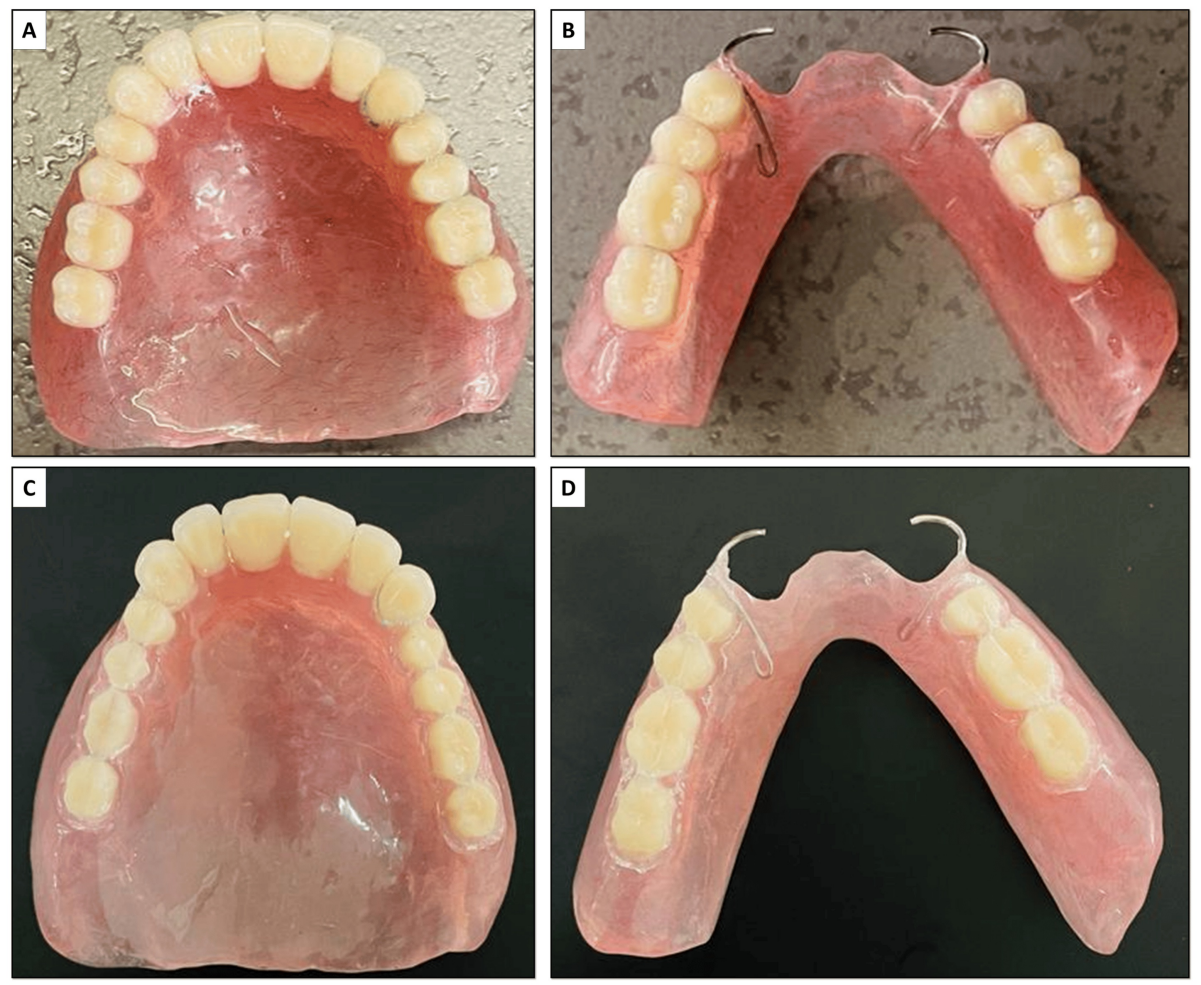
(A) and (B) Fabricated maxillary and mandibular dentures, respectively; (C) and D) Tooth preparation done on both dentures.

Jaw relation records were obtained and transferred using a facebow. These were used to mount casts with wax occlusal rims on a semi-adjustable articulator to simulate mandibular movements accurately (Figure 3). Anterior teeth selection was carried out based on the patient’s sex and personality, ensuring an aesthetically pleasing and natural appearance. Acrylic teeth (Biorock, Brulon International, Gundlav, Gujarat, India) of A2 shade were used for teeth arrangement. A balanced occlusion scheme was incorporated to distribute occlusal forces evenly and to prevent interferences during excursive mandibular movements, thereby enhancing chewing efficiency and comfort. After the try-in procedure (Figure 3), the denture was fabricated using heat-cured acrylic resin (Figure 4). The tooth preparation of premolars and molars within the dentures was performed using an airotor. A #13 tapered round bur was used to prepare the tooth for crowns with shoulder margins.

**Figure 5:**
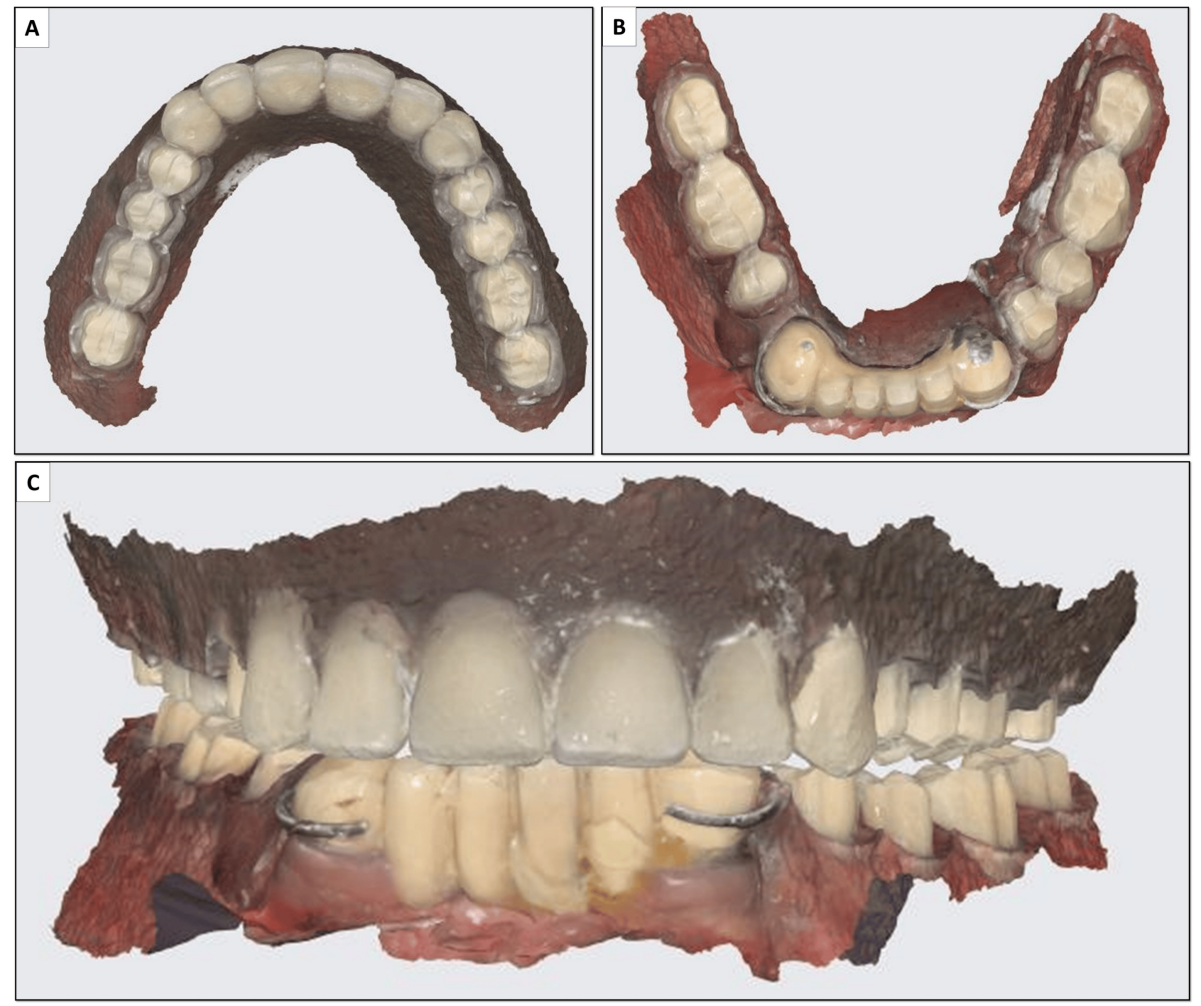
Digital scanning of (A) maxillary and (B) mandibular dentures after tooth preparation; (C) digital scan of both dentures in occlusion.

**Figure 6:**
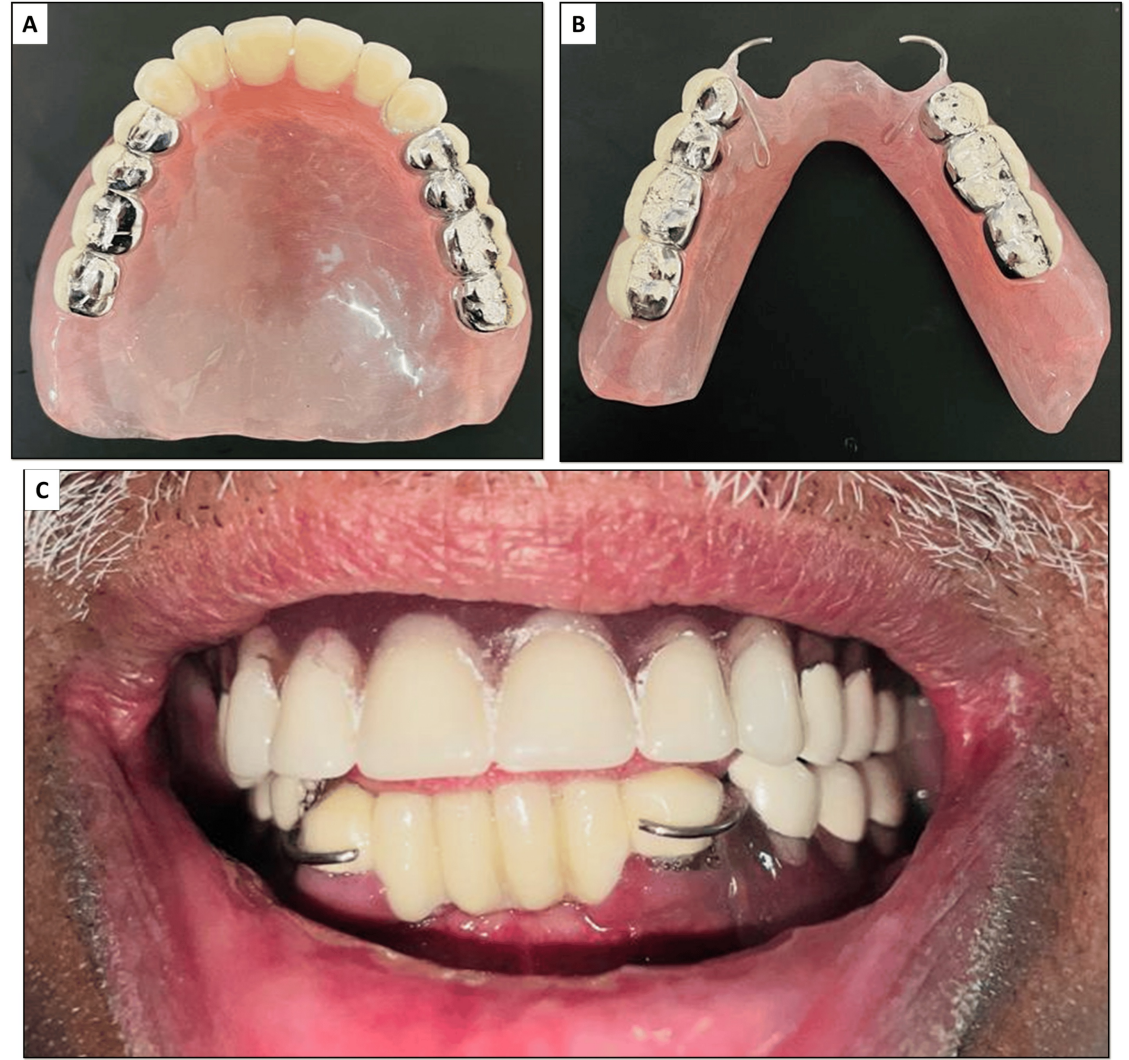
(A) Final look of the dentures after cementation of the DMLS crown on the dentures; (B) Intra-oral view after insertion of the dentures. DMLS: direct metal laser sintering

The completed denture was digitally scanned with the assistance of a specialized scanner (Medit i 600, Medit, Seoul, South Korea) and visualized using compatible software (Medit Link 3.3.2, Medit), as shown in Figure 5. The DMLS crowns were fabricated using a cobalt-chromium alloy based on the scan of the prepared teeth within the denture, specifically for the posterior teeth of both dentures. The DMLS crowns were subsequently cemented in place using glass ionomer cement (Figure 6). The dentures were adjusted for proper fit and occlusal adjustments were performed to ensure optimal functionality. Scanning the entire dentition before and after the tooth preparation helped in maintaining the vertical dimension for the patient.

**Figure 7:**
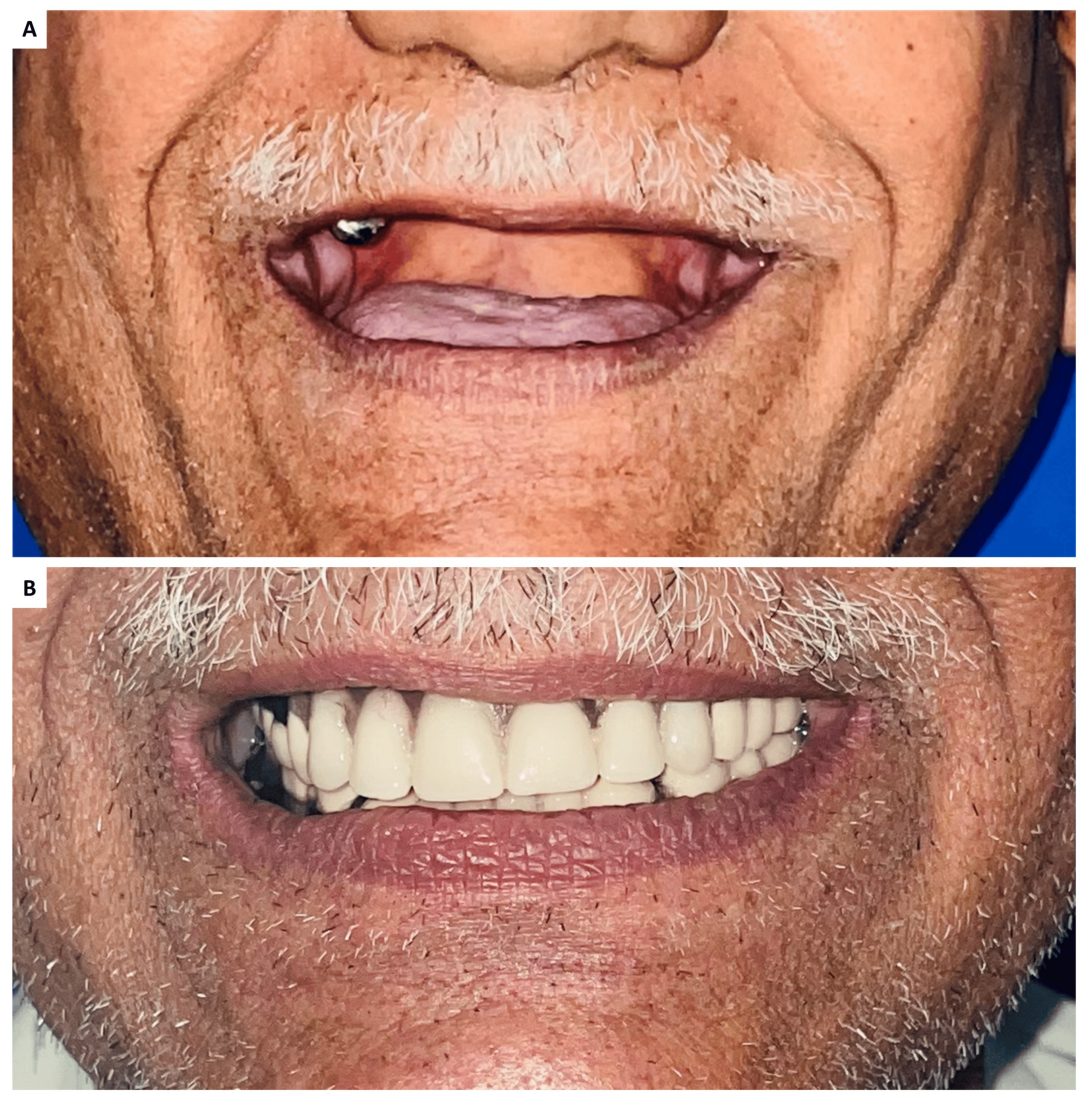
Comparison of aesthetic outcomes: (A) before and (B) after the insertion of dentures

The patient expressed satisfaction with the dentures from both aesthetic and functional perspectives (Figure 7). Comprehensive take-home instructions were provided as part of the maintenance phase therapy. The patient was educated and motivated to maintain oral hygiene, focusing on caries prevention and periodontal therapy to ensure a healthy oral environment. During a recall visit 12 months later, no complications or issues were observed, indicating successful treatment and patient compliance.

## Discussion

Rehabilitating patients with missing maxillary and lower posterior teeth using an upper complete denture and lower posterior partial denture is a daunting task in itself. Implant-supported dentures aid in retention and are, therefore, a preferred choice by clinicians [8,9]. However, because of non-feasibility, particularly in developing countries, many patients are unable to afford the treatment. Additionally, not all patients are eligible for multiple surgical procedures for implant placement. A conventional complete denture for the maxilla and distal-extension RPD for the mandible was thus, discerned as a more suitable treatment in the present case.

Preparation of these dentures requires careful planning in terms of balancing occlusal forces to avoid excessive loading at any area of the ridge. The treatment approach must, therefore, be customized to address the patient's specific issues. Application of excessive pressure on the anterior maxillary region while the posterior region has limited contact can further complicate the ridge resorption. Kelly reported a yearly alveolar bone loss of 0.43 mm in the anterior maxilla in such cases [2], which was further supported by a study by Lopez-Rolden et al., which found bone loss rates of 0.32 mm/year [10].

Maximizing the extension of denture bases and minimizing pressure on the anterior maxillary region can aid in controlling further bone loss [6]. Preservation of posterior teeth, partial extraction therapy, and the use of overdentures have been recommended. This was, however, not possible in the present case since the patient had already reported ill-fitting dentures and the teeth were already missing. To avoid disturbing the flabby anterior maxillary tissues, a mucostatic impression was used to capture the full denture-bearing area. Additionally, it has been demonstrated that less amount of bone resorption occurs in the maxillary anterior region when all the mandibular teeth are present as compared to the partially edentulous mandible, whether or not partial dentures are used [11]. Experimental studies have also suggested that patients with unilateral or bilateral mandibular RPDs experience greater bone resorption when opposing a complete denture in the maxilla [12]. Therefore, in contrast to the conventional RPDs, the occlusion must be meticulously planned in cases of combination syndrome. The impact of edentulous mandibles and retained mandibular teeth on the edentulous maxilla was thoroughly explored. The occlusal scheme was modified to ensure balanced occlusion across various mandibular movements. 

Attrition is a common issue in patients with combination syndrome [5,6]. Incorporating DMLS crowns into the denture design significantly reduces the attrition of denture teeth thereby preserving the occlusal harmony. The use of DMLS crowns enhances the durability and wear resistance of the denture teeth, preventing excessive wear and damage that can occur over time. This approach is the first of its kind to be attempted in the present case. We hypothesized it would help preserve the integrity of the prosthesis and prevent complications such as the breakage or deterioration of the denture teeth. By improving the longevity and stability of the denture, the risk of sequelae commonly associated with combination syndromes such as bone resorption or further prosthetic issues can be minimized.

## Conclusions

The rehabilitation of combination syndrome requires meticulous planning to address ridge resorption, occlusal imbalance, and functional limitations. This case demonstrated the successful use of a conventional maxillary complete denture and a mandibular removable partial denture, enhanced with DMLS crowns to improve occlusal stability and reduce denture tooth attrition. By optimizing force distribution and minimizing pressure on the anterior maxillary ridge, the treatment effectively mitigated further bone loss and provided long-term prosthetic stability. The 12-month follow-up confirmed functional and aesthetic success, reinforcing the efficacy of a well-planned, non-implant-based prosthetic approach for patients with financial or surgical constraints.
